# Origin of photovoltaic effect in superconducting YBa_2_Cu_3_O_6.96_ ceramics

**DOI:** 10.1038/srep11504

**Published:** 2015-06-23

**Authors:** F. Yang, M. Y. Han, F. G. Chang

**Affiliations:** 1 College of Physics and Electronic Engineering, Henan Normal University, Xinxiang 453007, P. R. China; 2 Henan Key Laboratory of Photovoltaic Materials, Xinxiang 453007, P. R. China

## Abstract

We report remarkable photovoltaic effect in YBa_2_Cu_3_O_6.96_ (YBCO) ceramic between 50 and 300 K induced by blue-laser illumination, which is directly related to the superconductivity of YBCO and the YBCO-metallic electrode interface. There is a polarity reversal for the open circuit voltage *V*_oc_ and short circuit current *I*_sc_ when YBCO undergoes a transition from superconducting to resistive state. We show that there exists an electrical potential across the superconductor-normal metal interface, which provides the separation force for the photo-induced electron-hole pairs. This interface potential directs from YBCO to the metal electrode when YBCO is superconducting and switches to the opposite direction when YBCO becomes nonsuperconducting. The origin of the potential may be readily associated with the proximity effect at metal-superconductor interface when YBCO is superconducting and its value is estimated to be ~10^–8^ mV at 50 K with a laser intensity of 502 mW/cm^2^. Combination of a p-type material YBCO at normal state with an n-type material Ag-paste forms a quasi-pn junction which is responsible for the photovoltaic behavior of YBCO ceramics at high temperatures. Our findings may pave the way to new applications of photon-electronic devices and shed further light on the proximity effect at the superconductor-metal interface.

Photo-induced voltage in high temperature superconductors has been reported in the early 1990’s and extensively investigated ever since, yet its nature and mechanism remain unsettled^1^,^2^,^3^,^4^,^5^. YBa_2_Cu_3_O_7-δ_ (YBCO) thin films^6^,^7^,^8^, in particular, are intensively studied in the form of photovoltaic (PV) cell due to its adjustable energy gap^9^,^10^,^11^,^12^,^13^. However, high resistance of the substrate always leads to a low conversion efficiency of the device and masks the primary PV properties of YBCO^8^. Here we report remarkable photovoltaic effect induced by blue-laser (λ = 450 nm) illumination in YBa_2_Cu_3_O_6.96_ (YBCO) ceramic between 50 and 300 K (*T*_c_ ~ 90 K). We show that the PV effect is directly related to the superconductivity of YBCO and the nature of the YBCO-metallic electrode interface. There is a polarity reversal for the open circuit voltage *V*_oc_ and short circuit current *I*_sc_ when YBCO undergoes a transition from superconducting phase to a resistive state. It is proposed that there exists an electrical potential across the superconductor-normal metal interface, which provides the separation force for the photo-induced electron-hole pairs. This interface potential directs from YBCO to the metal electrode when YBCO is superconducting and switches to the opposite direction when the sample becomes nonsuperconducting. The origin of the potential may be naturally associated with the proximity effect^14^,^15^,^16^,^17^ at metal-superconductor interface when YBCO is superconducting and its value is estimated to be ~10^−8^ mV at 50 K with a laser intensity of 502 mW/cm^2^. Combination of a p-type material YBCO at normal state with an n-type material Ag-paste forms, most likely, a quasi-pn junction which is responsible for the PV behavior of YBCO ceramics at high temperatures. Our observations shed further light on the origin of PV effect in high temperature superconducting YBCO ceramics and pave the way for its application in optoelectronic devices such as fast passive light detector etc.

## Results

**Figure 1:**
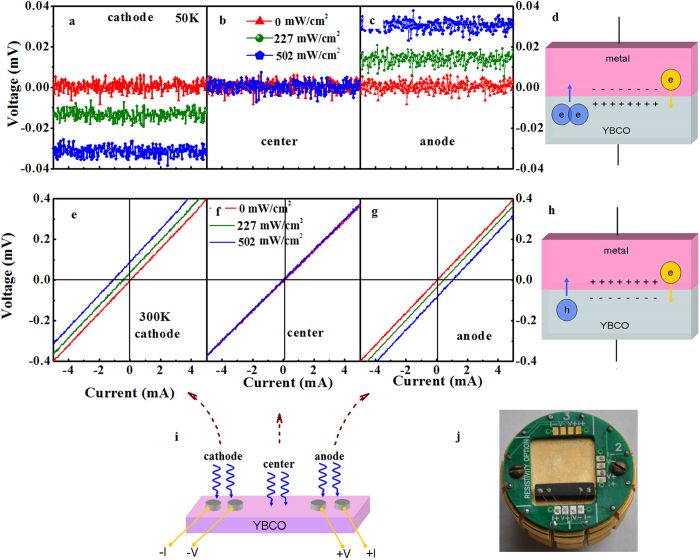
*I-V* characteristics of YBa_2_Cu_3_O_6.96_ illuminated by blue laser (λ = 450 nm) at 50 K (a–c) and 300 K (e–g). Values of *V*(*I*) were obtained by sweeping the current from −10 mA to +10 mA in vacuum. Only part of the experimental data is presented for the sake of clarity. **a**, Current-voltage characteristics of YBCO measured with laser spot positioned at the cathode (**i**). All the *I-V* curves are horizontal straight lines indicating the sample is still superconducting with laser irradiation. The curve moves down with increasing laser intensity, indicating that there exist a negative potential (*V*_oc_) between the two voltage leads even with zero current. The *I-V* curves remain unchanged when the laser is directed at the center of the sample at ether 50 K (**b**) or 300 K (**f**). The horizontal line moves up as the anode is illuminated (**c**). A schematic model of metal-superconductor junction at 50 K is shown in **d**. Current-voltage characteristics of normal state YBCO at 300 K measured with laser beam pointed at cathode and anode are given in **e** and **g** respectively. In contrast to the results at 50 K, non-zero slope of the straight lines indicates that YBCO is in normal state; the values of *V*_oc_ vary with light intensity in an opposite direction, indicating a different charge separation mechanism. A possible interface structure at 300 K is depicted in **h**. **j**. The real picture of the sample with leads.

Figure 1a–c shows that the *I-V* characteristics of YBCO ceramic sample at 50 K. Without light illumination, the voltage across the sample remains at zero with changing current, as can be expected from a superconducting material. Obvious photovoltaic effect appears when laser beam is directed at cathode (Fig. 1a): the *I-V* curves parallel to the *I*-axis moves downwards with increasing laser intensity. It is evident that there is a negative photo-induced voltage even without any current (often called open circuit voltage *V*_oc_). The zero slope of the *I-V* curve indicates that the sample is still superconducting under laser illumination.

Oxygen-rich YBCO in superconducting state can absorb almost full spectrum of sunlight due to its very small energy gap (*E*_g_)^9^,^10^, thereby creating electron-hole pairs (e–h). To produce an open circuit voltage *V*_oc_ by absorption of photons, it is necessary to spatially separate photo-generated e-h pairs before recombination occurs^18^. The negative *V*_oc,_ relative to the cathode and anode as indicated in Fig. 1i, suggests that there exists an electrical potential across the metal-superconductor interface, which sweeps the electrons to the anode and holes to the cathode. If this is the case, there should also be a potential pointing from superconductor to the metal electrode at anode. Consequently, a positive *V*_oc_ would be obtained if the sample area near the anode is illuminated. Furthermore, there should be no photo-induced voltages when the laser spot being pointed to areas far from the electrodes. It is certainly the case as can be seen from Fig. 1b,c!.

When the light spot moves from the cathode electrode to the center of the sample (about 1.25 mm apart from the interfaces), no variation of *I-V* curves and no *V*_oc_ can be observed with increasing laser intensity to the maximum value available (Fig. 1b). Naturally, this result can be ascribed to the limited lifetime of photo-induced carriers and the lack of separation force in the sample. Electron-hole pairs can be created whenever the sample is illuminated, but most of the e–h pairs will be annihilated and no photovoltaic effect is observed if the laser spot falls on areas far away from any of the electrodes. Moving the laser spot to the anode electrodes, the *I-V* curves parallel to the *I*-axis moves upwards with increasing laser intensity (Fig. 1c). Similar built-in electrical field exists in the metal-superconductor junction at the anode. However, the metallic electrode connects to the positive lead of the test system this time. The holes produced by the laser are pushed to the anode lead and thus a positive *V*_oc_ is observed. The results presented here provide strong evidence that there exists indeed an interface potential pointing from the superconductor to the metal electrode.

Photovoltaic effect in YBa_2_Cu_3_O_6.96_ ceramics at 300 K is shown in Fig. 1e–g. Without light illumination, *I-V* curve of the sample is a straight line crossing the origin. This straight line moves upwards parallel to the original one with increasing laser intensity irradiating at the cathode leads (Fig. 1e). There are two limiting cases of interest for a photovoltaic device. The short-circuit condition occurs when *V* = 0. The current in this case is referred to as the short circuit current (*I*_sc_). The second limiting case is the open-circuit condition (*V*_oc_) which occurs when *R*→∞ or the current is zero. Figure 1e clearly shows that *V*_oc_ is positive and increases with increasing light intensity, in contrast with the result obtained at 50 K; while a negative *I*_sc_ is observed to increase in magnitude with light illumination, a typical behavior of normal solar cells.

Similarly, when the laser beam is pointed at areas far away from the electrodes, the *V*(*I*) curve is independent of the laser intensity and there is no photovoltaic effect appeared (Fig. 1f). Similar to the measurement at 50 K, the *I-V* curves move to the opposite direction as the anode electrode is irradiated (Fig. 1g). All these results obtained for this YBCO-Ag paste system at 300 K with laser irradiated at different positions of the sample are consistent with an interface potential opposite to that observed at 50 K.

## Discussion

Most of electrons condense in Cooper pairs in superconducting YBCO below its transition temperature *T*_c_. While in the metal electrode, all the electrons remain in singular form. There is a large density gradient for both singular electrons and Cooper pairs in the vicinity of the metal-superconductor interface. Majority-carrier singular electrons in metallic material will diffuse into the superconductor region, whereas majority-carrier Cooper-pairs in YBCO region will diffuse into the metal region. As Cooper pairs carrying more charges and having a larger mobility than singular electrons diffuse from YBCO into metallic region, positively charged atoms are left behind, resulting in an electric field in the space charge region. The direction of this electric field is shown in the schematic diagram Fig. 1d. Incident photon illumination near the space charge region can create e-h pairs that will be separated and swept out producing a photocurrent in the reverse-bias direction. As soon as the electrons get out of the build-in electrical field, they are condensed into pairs and flow to the other electrode without resistance. In this case, the *V*_oc_ is opposite to the pre-set polarity and displays a negative value when the laser beam points to the area around the negative electrode. From the value of *V*_oc_, the potential across the interface can be estimated: the distance between the two voltage leads *d* is ~5 × 10^−3^ m, the thickness of the metal-superconductor interface, *d*_*i*_, should be the same order of magnitude as the coherence length of YBCO superconductor (~1 nm)^19^,^20^, take the value of *V*_oc_ = 0.03 mV, the potential *V*_ms_ at the metal-superconductor interface is evaluated to be ~10^−11^ V at 50 K with a laser intensity of 502 mW/cm^2^, using equation,


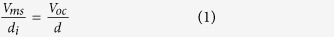


We want to emphasize here that the photo-induced voltage cannot be explained by photo thermal effect. It has been experimentally established that the Seebeck coefficient of superconductor YBCO is *S*_s_ = 0^21^. The Seebeck coefficient for copper lead wires is in the range of *S*_Cu_ = 0.34–1.15 μV/K^3^. The temperature of the copper wire at the laser spot can be raised up by a small amount of 0.06 K with maximum laser intensity available at 50 K. This could produce a thermoelectric potential of 6.9 × 10^−8^ V which is three orders magnitude smaller than the *V*_oc_ obtained in Fig 1 (a). It is evident that thermoelectric effect is too small to explain the experimental results. In fact, the temperature variation due to laser irradiation would disappear in less than one minute so that the contribution from thermal effect can be safely ignored.

This photovoltaic effect of YBCO at room temperature reveals that a different charge separation mechanism is involved here. Superconducting YBCO in normal state is a p-type material with holes as charge carrier^22^,^23^, while metallic Ag-paste has characteristics of an n-type material. Similar to p-n junctions, the diffusion of electrons in the silver paste and holes in YBCO ceramic will form an internal electrical field pointing to the YBCO ceramic at the interface (Fig. 1h). It is this internal field that provides the separation force and leads to a positive *V*_oc_ and negative *I*_sc_ for the YBCO-Ag paste system at room temperature, as shown in Fig. 1e. Alternatively, Ag-YBCO could form a p-type Schottky junction which also leads to an interface potential with the same polarity as in the model presented above^24^.

**Figure 2:**
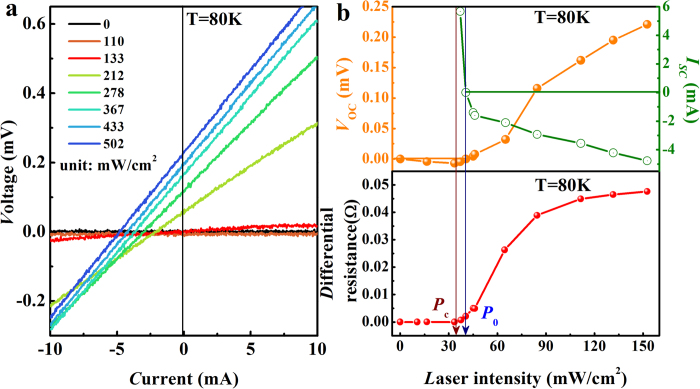
Photovoltaic characteristics as a function of laser intensity for YBCO-Ag paste system at 80 K. The laser spot center is positioned around the cathode electrodes (see Fig. 1i). **a**, *I-V* curves of YBCO irradiated with different laser intensities. **b** (top), Laser intensity dependence of open circuit voltage *V*_oc_ and short circuit current *I*_sc_. The *I*_sc_ values can not be obtained at low light intensity (* < *110 mW/cm^2^) because the *I-V* curves are parallel to the *I*-axis when the sample is in superconducting state. **b** (bottom), differential resistance as a function of laser intensity.

To investigate the detailed evolution process of the photovoltaic properties during superconducting transition of YBCO, *I-V* curves of the sample at 80 K were measured with selected laser intensities illuminating at cathode electrode (Fig. 2). Without laser irradiation, the voltage across the sample keeps at zero regardless of current, indicating the superconducting state of the sample at 80 K (Fig. 2a). Similar to the data obtained at 50 K, *I-V* curves parallel to the *I*-axis moves downwards with increasing laser intensity until a critical value *P*_c_ is reached. Above this critical laser intensity (*P*_c_), the superconductor undergoes a transition from a superconducting phase to a resistive phase; the voltage starts to increase with current due to the appearance of resistance in the superconductor. As a result, the *I-V* curve begins to intersect with the *I*-axis and *V*-axis leading to a negative *V*_oc_ and a positive *I*_sc_ at first. Now the sample seems to be in a special state in which the polarity of *V*_oc_ and *I*_sc_ is extremely sensitive to light intensity; with very small increase in light intensity *I*_sc_ is converted from positive to negative and *V*_oc_ from negative to positive value, passing the origin (the high sensitivity of photovoltaic properties, particularly the value of *I*_sc_, to light illumination can be seen more clearly in Fig. 2b). At the highest laser intensity available, the *I-V* curves intend to be parallel with each other, signifying the normal state of the YBCO sample.

The laser intensity dependence of *V*_oc_ and *I*_sc_ at 80 K is shown in Fig. 2b (top). The photovoltaic properties can be discussed in three regions of light intensity. The first region is between 0 and *P*_c_, in which YBCO is superconducting, *V*_oc_ is negative and decreases (absolute value increases) with light intensity and reaching a minimum at *P*_c_. The second region is from *P*_c_ to another critical intensity *P*_0_, in which *V*_oc_ increases while *I*_sc_ decreases with increasing light intensity and both reach zero at *P*_0_. The third region is above *P*_0_ until normal state of YBCO is reached. Although both *V*_oc_ and *I*_sc_ vary with light intensity in the same way as in region 2, they have opposite polarity above the critical intensity *P*_0_. The significance of *P*_0_ lies in that there is no photovoltaic effect and the charge separation mechanism changes qualitatively at this particular point. The YBCO sample becomes non-superconducting in this range of light intensity but the normal state yet to be reached.

Clearly, the photovoltaic characteristics of the system are closely related to the superconductivity of YBCO and its superconducting transition. The differential resistance, *dV/dI*, of YBCO is shown in Fig. 2b (bottom) as a function of laser intensity. As mentioned before, the build-in electric potential in the interface due to Cooper pair diffusion points from the superconductor to metal. Similar to that observed at 50 K, photovoltaic effect is enhanced with increasing laser intensity from 0 to *P*_c_. When the laser intensity reaches a value slightly above *P*_c_, the *I-V* curve starts to tilt, and the resistance of the sample begins to appear, but the polarity of the interface potential is not changed yet. The effect of optical excitation on the superconductivity has been investigated in the visible or near-IR region. While the basic process is to break up the Cooper pairs and destroy the superconductivity^25^,^26^, in some cases superconductivity transition can be enhanced^27^,^28^,^29^, new phases of superconductivity can even be induced^30^. The absence of superconductivity at *P*_c_ can be ascribed to the photo-induced pair breaking. At the point *P*_0_, the potential across the interface becomes zero, indicating the charge density in both sides of the interface reaches the same level under this particular intensity of light illumination. Further increase in laser intensity results in more Cooper pairs being destroyed and YBCO is gradually transformed back to a p-type material. Instead of electron and Cooper pair diffusion, the feature of the interface is now determined by electron and hole diffusion which leads to a polarity reversal of the electrical field in the interface and consequently a positive *V*_oc_ (compare Fig.1d,h). At very high laser intensity, the differential resistance of YBCO saturates to a value corresponding to the normal state and both *V*_oc_ and *I*_sc_ tend to vary linearly with laser intensity (Fig. 2b). This observation reveals that laser irradiation on normal state YBCO will no longer change its resistivity and the feature of the superconductor-metal interface but only increase the concentration of the electron-hole pairs.

**Figure 3:**
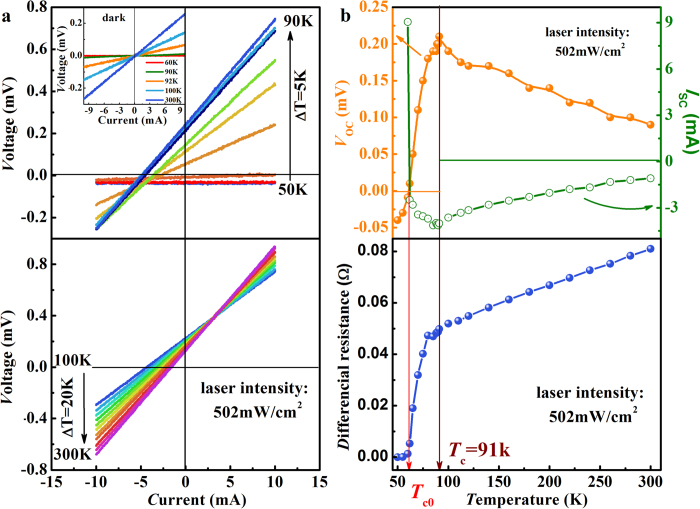
Temperature dependence of photovoltaic characteristics for YBCO-Ag paste system under 502 mW/cm^2^ laser illumination. The laser spot center is positioned around the cathode electrodes (see Fig. 1i). **a**, *I-V* curves obtained from 50 to 90 K (top) and from 100 to 300 K (bottom) with a temperature increment of 5 K and 20 K, respectively. Inset **a** shows *I-V* characteristics at several temperatures in dark. All the curves cross the origin point. **b**, open circuit voltage *V*_oc_ and short circuit current *I*_sc_ (top) and the differential resistance, *dV*/*dI*, of YBCO (bottom) as a function of temperature. The zero resistance superconducting transition temperature *T*_cp_ is not given because it is too close to *T*_c0_.

To investigate the effect of temperature on the photovoltaic properties, the metal-superconductor system was irradiated at the cathode with blue laser of intensity 502 mW/cm^2^. *I-V* curves obtained at selected temperatures between 50 and 300 K are given in Fig. 3a. The open circuit voltage *V*_oc_, short circuit current *I*_sc_ and the differential resistance can then be obtained from these *I-V* curves and are shown in Fig. 3b. Without light illumination, all the *I-V* curves measured at different temperatures pass the origin as expected (inset of Fig. 3a). The *I-V* characteristics change drastically with increasing temperature when the system is illuminated by a relatively strong laser beam (502 mW/cm^2^). At low temperatures the *I-V* curves are straight lines parallel to the *I*-axis with negative values of *V*_oc_. This curve moves upwards with increasing temperature, and gradually turns into a line with a nonzero slope at a critical temperature *T*_cp_ (Fig. 3a (top)). It seems that all the *I-V* characteristic curves rotate around a point in the third quadrant. *V*_oc_ increases from a negative value to a positive one while *I*_sc_ decreases from a positive to a negative value. Above the original superconducting transition temperature *T*_c_ of YBCO, the *I-V* curve changes rather differently with temperature (bottom of Fig. 3a). Firstly, the rotation center of the *I-V* curves moves to the first quadrant. Secondly, *V*_oc_ keeps decreasing and *I*_sc_ increasing with increasing temperature (top of Fig. 3b). Thirdly, the slope of the *I-V* curves increase linearly with temperature resulting in a positive temperature coefficient of resistance for YBCO (bottom of Fig. 3b).

Three critical temperatures can be recognized from Fig. 3b: *T*_cp_, above which YBCO becomes non-superconducting; *T*_c0_, at which both *V*_oc_ and *I*_sc_ become zero and *T*_c_, the original onset superconducting transition temperature of YBCO without laser irradiation. Below *T*_cp_ ~ 55 K, the laser irradiated YBCO is in superconducting state with relatively high concentration of Cooper pairs. The effect of laser irradiation is to reduce the zero resistance superconducting transition temperature from 89 K to ~55 K (bottom of Fig. 3b) by reducing the Cooper pair concentration in addition to producing photovoltaic voltage and current. Increasing temperature also breaks down the Cooper pairs leading to a lower potential in the interface. Consequently, the absolute value of *V*_oc_ will become smaller, although same intensity of laser illumination is applied. The interface potential will become smaller and smaller with further increase in temperature and reaches zero at *T*_c0_. There is no photovoltaic effect at this special point because there is no internal field to separate the photo-induced electron-hole pairs. A polarity reversal of the potential occurs above this critical temperature as the free charge density in Ag paste is greater than that in YBCO which is gradually transferred back to a p-type material. Here we want to emphasize that the polarity reversal of *V*_oc_ and *I*_*sc*_ occurs immediately after the zero resistance superconducting transition, regardless of the cause of the transition. This observation reveals clearly, for the first time, the correlation between superconductivity and the photovoltaic effects associated with the metal-superconductor interface potential. The nature of this potential across the superconductor-normal metal interface has been a research focus for the last several decades but there are many questions still waiting to be answered. Measurement of the photovoltaic effect may prove to be an effective method for exploring the details (such as its strength and polarity etc.) of this important potential and hence shed light on the high temperature superconducting proximity effect.

Further increase in temperature from *T*_c0_ to *T*_c_ leads to a smaller concentration of Cooper pairs and an enhancement in the interface potential and consequently larger *V*_oc_. At *T*_c_ the Cooper pair concentration becomes zero and the build-in potential at the interface reaches a maximum, resulting in maximum *V*_oc_ and minimum *I*_sc_. The rapid increase of *V*_oc_ and *I*_sc_ (absolute value) in this temperature range corresponds to the superconducting transition which is widened from *ΔT* ~ 3 K to ~34 K by laser irradiation of intensity 502 mW/cm^2^ (Fig. 3b). In the normal states above *T*_c_, the open circuit voltage *V*_oc_ decreases with temperature (top of Fig. 3b), similar to the linear behavior of *V*_oc_ for normal solar cells based on p-n junctions^31^,^32^,^33^. Although the change rate of *V*_oc_ with temperature (−*dV*_oc_*/dT*), which depends strongly on laser intensity, is much smaller than that of normal solar cells, the temperature coefficient of *V*_oc_ for YBCO-Ag junction has the same order of magnitude as that of the solar cells. The leakage current of a p-n junction for a normal solar cell device increases with increasing temperature, leading to a decrease in *V*_oc_ as temperature increases. The linear *I-V* curves observed for this Ag-superconductor system, due to firstly the very small interface potential and secondly the back-to-back connection of the two heterojunctions, makes it difficult to determine the leakage current. Nevertheless, it seams very likely that the same temperature dependence of leakage current is responsible for the *V*_oc_ behaviour observed in our experiment. According to the definition, *I*_sc_ is the current needed to produce a negative voltage to compensate *V*_oc_ so that the total voltage is zero. As temperature increases, *V*_oc_ becomes smaller so that less current is needed to produce the negative voltage. Furthermore, the resistance of YBCO increases linearly with temperature above *T*_c_ (bottom of Fig. 3b), which also contributes to the smaller absolute value of *I*_sc_ at high temperatures.

Notice that the results given in Figs 2,3 are obtained by laser irradiating at the area around cathode electrodes. Measurements have also been repeated with laser spot positioned at anode and similar *I-V* characteristics and photovoltaic properties have been observed except that the polarity of *V*_oc_ and *I*_*sc*_ has been reversed in this case. All these data lead to a mechanism for the photovoltaic effect, which is closely related to the superconductor-metal interface.

In summary, the *I-V* characteristics of laser irradiated superconducting YBCO-Ag paste system have been measured as functions of temperature and laser intensity. Remarkable photovoltaic effect has been observed in the temperature range from 50 to 300 K. It is found that the photovoltaic properties correlate strongly to the superconductivity of YBCO ceramics. A polarity reversal of *V*_oc_ and *I*_sc_ occurs immediately after the photo-induced superconducting to non-superconducting transition. Temperature dependence of *V*_oc_ and *I*_sc_ measured at fixed laser intensity shows also a distinct polarity reversal at a critical temperature above which the sample becomes resistive. By locating the laser spot to different part of the sample, we show that there exists an electrical potential across the interface, which provides the separation force for the photo-induced electron-hole pairs. This interface potential directs from YBCO to the metal electrode when YBCO is superconducting and switches to the opposite direction when the sample becomes nonsuperconducting. The origin of the potential may be naturally associated with the proximity effect at metal-superconductor interface when YBCO is superconducting and is estimated to be ~10^−8^ mV at 50 K with a laser intensity of 502 mW/cm^2^. Contact of a p-type material YBCO at normal state with a n-type material Ag-paste forms a quasi-pn junction which is responsible for the photovoltaic behavior of YBCO ceramics at high temperatures. The above observations shed light on the PV effect in high temperature superconducting YBCO ceramics and pave the way to new applications in optoelectronic devices such as fast passive light detector and single photon detector.

## Methods

The photovoltaic effect experiments were performed on an YBCO ceramic sample of 0.52 mm thickness and 8.64 × 2.26 mm^2^ rectangular shape and illuminated by continuous wave blue-laser (λ = 450 nm) with laser spot size of 1.25 mm in radius. Using bulk rather than thin film sample enables us to study the photovoltaic properties of the superconductor without having to deal with the complex influence of the substrate^6^,^7^. Moreover, the bulk material could be conducive for its simple preparation procedure and relatively low cost. The copper lead wires are cohered on the YBCO sample with silver paste forming four circular electrodes about 1 mm in diameter. The distance between the two voltage electrodes is about 5 mm. *I-V* characteristics of the sample were measured using the vibration sample magnetometer (VersaLab, Quantum Design) with a quartz crystal window. Standard four-wire method was employed to obtain the *I-V* curves. The relative positions of electrodes and the laser spot are shown in Fig. 1i.

## Additional Information

**How to cite this article**: Yang, F. *et al.* Origin of photovoltaic effect in superconducting YBa_2_Cu_3_O_6.96_ ceramics. *Sci. Rep.* **5**, 11504; doi: 10.1038/srep11504 (2015).

## References

[b1] Chang C. L., Kleinhammes A., Moulton W. G. & Testardi L. R. Symmetry-forbidden laser-induced voltages in YBa_2_Cu_3_O_7_. Phys. Rev. B 41, 11564–11567 (1990). 10.1103/physrevb.41.11564 9993578

[b2] Kwok H. S., Zheng J. P. & Dong S. Y. Origin of the anomalous photovoltaic signal in Y-Ba-Cu-O. Phys. Rev. B 43, 6270–6272 (1991). 10.1103/physrevb.43.6270 9998058

[b3] Wang L. P., Lin J. L., Feng Q. R. & Wang G. W. Measurement of laser-induced voltages of superconducting Bi-Sr-Ca-Cu-O. Phys. Rev. B 46, 5773–5776 (1992). 10.1103/physrevb.46.5773 10004377

[b4] Tate K. L., *et al.* Transient laser-induced voltages in room-temperature films of YBa_2_Cu_3_O_7-x_. J. Appl. Phys. 67, 4375–4376 (1990).

[b5] Kwok H. S. & Zheng J. P. Anomalous photovoltaic response in YBa_2_Cu_3_O_7_. Phys. Rev. B 46, 3692–3695 (1992). 10.1103/physrevb.46.3692 10004091

[b6] Muraoka Y., Muramatsu T., Yamaura J. & Hiroi Z. Photogenerated hole carrier injection to YBa_2_Cu_3_O_7−x_ in an oxide heterostructure. Appl. Phys. Lett. 85, 2950–2952 (2004).

[b7] Asakura D. *et al.* Photoemission study of YBa_2_Cu_3_O_y_ thin films under light illumination. Phys. Rev. Lett. 93, 247006 (2004). 15697853 10.1103/PhysRevLett.93.247006

[b8] Yang F. *et al.* Photovoltaic effect of YBa_2_Cu_3_O_7-δ_/SrTiO_3_ :Nb heterojunction annealed in different oxygen partial pressure. Mater. Lett. 130, 51–53 (2014).

[b9] Aminov B. A. *et al.* Two-Gap structure in Yb(Y)Ba_2_Cu_3_O_7-x_ single crystals. J. Supercond. 7, 361–365 (1994).

[b10] Kabanov V. V., Demsar J., Podobnik B. & Mihailovic D. Quasiparticle relaxation dynamics in superconductors with different gap structures: Theory and experiments on YBa_2_Cu_3_O_7-δ_. Phys. Rev. B 59, 1497–1506 (1999).

[b11] Sun J. R., Xiong C. M., Zhang Y. Z. & Shen B. G. Rectifying properties of the YBa_2_Cu_3_O_7-δ_/SrTiO_3_ :Nb heterojunction. Appl. Phys. Lett. 87, 222501 (2005).

[b12] Kamarás K., Porter C. D., Doss M. G., Herr S. L. & Tanner D. B. Excitonic absorption and superconductivity in YBa_2_Cu_3_O_7-δ_. Phys. Rev. Lett. 59, 919–922 (1987). 10035906 10.1103/PhysRevLett.59.919

[b13] Yu G., Heeger A. J. & Stucky G. Transient photoinduced conductivity in semiconducting single crystals of YBa_2_Cu_3_O_6.3_: search for photoinduced metallic state and for photoinduced superconductivity. Solid State Commun. 72, 345–349 (1989).

[b14] McMillan W. L. Tunneling model of the superconducting proximity effect. Phys. Rev. 175, 537–542 (1968).

[b15] Guéron S. *et al.* Superconducting proximity effect probed on a mesoscopic length scale. Phys. Rev. Lett. 77, 3025–3028 (1996). 10062112 10.1103/PhysRevLett.77.3025

[b16] Annunziata G. & Manske D. Proximity effect with noncentrosymmetric superconductors. Phys. Rev. B 86, 17514 (2012).

[b17] Qu F. M. *et al.* Strong superconducting proximity effect in Pb-Bi_2_Te_3_ hybrid structures. Sci. Rep. 2, 339 (2012). 22468226 10.1038/srep00339 PMC3314303

[b18] Chapin D. M., Fuller C. S. & Pearson G. L. A new silicon p-n junction photocell for converting solar radiation into electrical power. J. App. Phys. 25, 676–677 (1954).

[b19] Tomimoto K. Impurity effects on the superconducting coherence length in Zn- or Ni-doped YBa_2_Cu_3_O_6.9_ single crystals. Phys. Rev. B 60, 114–117 (1999).

[b20] Ando Y. & Segawa K. Magnetoresistance of Untwinned YBa_2_Cu_3_O_y_ single crystals in a wide range of doping: anomalous hole-doping dependence of the coherence length. Phys. Rev. Lett. 88, 167005 (2002). 11955252 10.1103/PhysRevLett.88.167005

[b21] Obertelli S. D. & Cooper J. R. Systematics in the thermoelectric power of high-T, oxides. Phys. Rev. B 46, 14928–14931, (1992). 10.1103/physrevb.46.14928 10003604

[b22] Sugai S. *et al.* Carrier-density-dependent momentum shift of the coherent peak and the LO phonon mode in p-type high-*T*_c_ superconductors. Phys. Rev. B 68, 184504 (2003).

[b23] Nojima T. *et al.* Hole reduction and electron accumulation in YBa_2_Cu_3_O_y_ thin films using an electrochemical technique: Evidence for an n-type metallic state. Phys. Rev. B 84, 020502 (2011).

[b24] Tung R. T. The physics and chemistry of the Schottky barrier height. Appl. Phys. Lett. 1, 011304 (2014).

[b25] Testardi I. R. Destruction of superconductivity by laser light. Phys. Rev. B 4, 2189–2196 (1971).

[b26] Sai-Halasz G. A., Chi C. C., Denenstein A. & Langenberg D. N. Effects of Dynamic External Pair Breaking in Superconducting Films. Phys. Rev. Lett. 33, 215–219 (1974).

[b27] Nieva G. *et al.* Photoinduced enhancement of superconductivity. Appl. Phys. Lett. 60, 2159–2161 (1992).

[b28] Kudinov V. I. *et al.* Persistent photoconductivity in YBa_2_Cu_3_O_6+x_ films as a method of photodoping toward metallic and superconducting phases. Phys. Rev. B 14, 9017–9028 (1993). 10.1103/physrevb.47.9017 10004951

[b29] Mankowsky R. *et al.* Nonlinear lattice dynamics as a basis for enhanced superconductivity in YBa_2_Cu_3_O_6.5_. Nature 516, 71–74 (2014). 25471882 10.1038/nature13875

[b30] Fausti D. *et al.* Light-induced superconductivity in a stripe-ordered cuprate. Science 331, 189–191 (2011). 21233381 10.1126/science.1197294

[b31] El-Adawi M. K. & Al-Nuaim I. A. The temperature functional dependence of VOC for a solar cell in relation to its efficiency new approach. Desalination 209, 91–96 (2007).

[b32] Vernon S. M. & Anderson W. A. Temperature effects in Schottky-barrier silicon solar cells. Appl. Phys. Lett. 26, 707 (1975).

[b33] Katz E. A., Faiman D. & Tuladhar S. M. Temperature dependence for the photovoltaic device parameters of polymer-fullerene solar cells under operating conditions. J. Appl. Phys. 90, 5343–5350 (2002).

